# Evaluating the impact of first-yes-counts instructions on eyewitness performance using the two-high threshold eyewitness identification model

**DOI:** 10.1038/s41598-023-33424-4

**Published:** 2023-04-21

**Authors:** Kristina Winter, Nicola Marie Menne, Raoul Bell, Axel Buchner

**Affiliations:** Department of Experimental Psychology, Heinrich Heine University Düsseldorf, 40204 Düsseldorf, Germany

**Keywords:** Psychology, Human behaviour

## Abstract

In eyewitness research, multiple identification decisions in sequential lineups are typically prevented by telling participants that only their first identification decision counts. These first-yes-counts instructions are incompatible with standard police protocols prescribing that witnesses shall see the entire lineup. Horry et al. were the first to experimentally test how this discrepancy between eyewitness research and standard police protocols affects eyewitness identification decisions. Here, the two-high threshold eyewitness identification model was used to disentangle the effect of the first-yes-counts instructions on the detection and guessing processes underlying eyewitness identification decisions. We report both a reanalysis of Horry et al.’s data and a conceptual replication. Both the reanalysis and the results of the conceptual replication confirm that first-yes-counts instructions do not affect the detection of the culprit but decrease the probability of guessing-based selections. To improve the ecological validity, research on sequential lineups should avoid first-yes-counts instructions.

Following science-based recommendations (e.g.,^1^), many jurisdictions have replaced simultaneous lineups in which eyewitnesses see photographs of a single suspect and a finite number of fillers at once with sequential lineups (e.g.,^2–6^). In a typical sequential lineup, photographs of one suspect and a finite number of fillers are shown in a random sequence and an eyewitness makes an identification decision or a non-identification decision for each photograph separately. If an eyewitness makes only non-identification decisions, the lineup is rejected. At the other extreme, the sequential presentation implies, at least in principle, that an eyewitness can make as many identification decisions as there are photographs in the lineup. In eyewitness research, multiple identification decisions in sequential lineups are typically prevented by first-yes-counts instructions^7,8^. In these instructions, participants are informed that only their first identification decision counts. Sometimes, the lineup presentation continues after a first identification decision has been made and participants are shown the complete lineup (e.g.,^9–17^). In other studies, lineup presentation is terminated immediately after the first identification decision (e.g.,^18–27^). In sharp contrast, standard police protocols do not use first-yes-counts instructions^2–5,8,28–31^. Instead, lineup presentation continues after identification decisions and eyewitnesses can select further photographs if these provide a better match to the memory of the culprit. Given this discrepancy between research and police practice, it is important to understand the effects of first-yes-counts instructions on the processes involved in eyewitness identification decisions.

Horry et al.^32^ were the first to evaluate this discrepancy experimentally. Among other things, they compared a sequential-lineup condition in which participants were told that only their first yes-response would count as an identification decision to a sequential-lineup condition without first-yes-counts instructions. The suspect identification rate was numerically but not significantly lower (0.26 compared to 0.29) and the lineup rejection rate was significantly higher (0.53 compared to 0.43) in the condition with first-yes-counts instructions compared to the condition without such instructions. This result was interpreted to indicate that first-yes-counts instructions increase participants’ response criterion. Such an interpretation seems plausible given that participants in the condition with first-yes-counts instructions knew that they had only one chance to make an identification decision. These participants may have been particularly hesitant to select a lineup member, not knowing whether another lineup member would follow which might be an even better match to their memory of the culprit and to which they would then no longer be able to make an identification decision. Whereas two response rates were used by Horry et al.^32^ as a basis for drawing conclusions about participants’ response criterion, receiver operating characteristics were used as a measurement model to assess whether the instructions affected participants’ ability to discriminate the culprit from the innocent suspect. In terms of the partial area under the curve, there was a numerical, but not statistically significant, difference between the sequential-lineup condition with first-yes-counts instructions (partial area under the curve = 0.014) and the sequential-lineup condition without such instructions (partial area under the curve = 0.017). Based on these two types of analyses, it is possible to draw the conclusions that first-yes-counts instructions induce a conservative response bias while leaving the overall ability to discriminate the culprit from the innocent suspect unaffected.

If these conclusions are valid, then they have important implications in that they suggest that a sizeable number of previous eyewitness identification studies may lack ecological validity. Specifically, if first-yes-counts instructions affect the processes underlying eyewitness identification decisions, inferences based on data from laboratory studies using first-yes-counts instructions may not apply to lineups that follow standard police protocols. For instance, sequential lineups have been associated with particularly conservative responding^7^ but this finding may only be an artifact of first-yes-counts instructions. However, a necessary condition for the validity of these conclusions is that the findings on which they are based are reliable^33^. Therefore, an independent replication of Horry et al.’s^32^ sequential-lineup conditions is needed before drawing such far-reaching conclusions.

Independent of the question of reliability, there are at least three threats to the validity of Horry et al.’s^32^ conclusions. First, Horry et al.^32^ used two separate types of response rates—the suspect identification rate and the lineup rejection rate—which they implicitly combined in their conclusion about the effect of first-yes-counts instructions into a two-component response-bias measure, albeit a measure that is not readily quantifiable. At this point it is important to realize that there is no such thing as a measure without a measurement model. This means that by implicitly combining the suspect identification rate and the lineup rejection rate into a (not readily quantifiable) two-component response-bias measure, an ad-hoc measurement model was applied. In a verbal approximation, this measurement model might look something like this: A decreasing suspect identification rate and an increasing lineup rejection rate may be assumed to indicate a decreasing willingness to make an identification decision which may be seen as an index of an increasingly conservative response bias. This measurement model may seem intuitively plausible and thus may have some face validity. Nevertheless, it is preferable to rely on a measurement model that is not only intuitively plausible but one that has also been validated experimentally, that is, for which it has been demonstrated experimentally that it really measures the processes it is intended to measure. To anticipate, here we will use such a validated measurement model. Second, the effect of first-yes-counts instructions on the implicit, not readily quantifiable response-criterion measure was assessed by performing two separate statistical tests of which one showed no significant difference between the instruction conditions whereas the other did. Specifically, the suspect identification rate was only numerically but not significantly lower in the condition with first-yes-counts instructions compared to the condition without such instructions while the lineup rejection rate was significantly higher in the condition with first-yes-counts instructions compared to the condition without such instructions. The fact that only one of the two tests was statistically significant creates some ambiguity as to the difference in response bias between the instruction conditions: According to the first test with a nonsignificant result one has to conclude that there is no difference in response bias between the instruction conditions whereas according to the second test with a statistically significant result one has to conclude that such a difference in response bias between test conditions exists. To arrive at unambiguous conclusions, it is better to have a single measure that can be compared between conditions using a single statistical test. To anticipate, the measurement model we use here provides such a measure. Third, a completely different measurement model based on receiver operating characteristics was used for assessing the effect of the first-yes-counts instructions on the ability to discriminate the culprit from the innocent suspect. Instead of relying on two entirely different measurement models with different sets of underlying assumptions, it seems desirable to use one single measurement model based on one coherent set of assumptions for measuring various types of processes underlying eyewitness identification decisions. Ideally it should also be possible to demonstrate that this measurement model fits the data before measures are interpreted.

The two-high threshold (2-HT) eyewitness identification model is such a measurement model. The model has been experimentally validated: Multiple experiments have shown that the model’s measures indeed reflect the processes they were designed to measure^34,35^. The 2-HT eyewitness identification model belongs to the class of multinomial processing tree models^36–38^. These models are used to provide estimates of measures representing the probabilities of latent processes from categorical data. Statistical hypotheses are tested directly at the level of these parameters. Based on the entire 2 × 3 data structure of eyewitness identification decisions (i.e., correct culprit identifications, false innocent-suspect identifications, false filler identifications in culprit-present and culprit-absent lineups, correct and false lineup rejections), the 2-HT eyewitness identification model provides measures of the detection of the presence of the culprit (*dP*) and of the detection of the absence of the culprit in the lineup (*dA*), a measure of the biased selection of the suspect in unfair lineups (*b*) and a measure of selections based on guessing processes (*g*).

Figure 1 shows a graphical illustration of the model. The upper tree represents the processes leading to culprit and filler identifications as well as lineup rejections in culprit-present lineups. The eyewitness detects the presence of the culprit with probability *dP*. If culprit detection fails, which occurs with probability 1 – *dP*, then the eyewitness may identify the culprit based on two types of non-detection-based processes. If the lineup is unfair in that the culprit stands out from the fillers so that it can be inferred who the culprit is without relying on memory, then biased selection of the culprit occurs with probability *b*. With probability 1 – *b*, no biased selection of the culprit occurs (e.g., if the lineup is fair or the eyewitness does not attend to the features creating the unfairness or chooses to ignore these features). In this case, it is still possible to select one of the lineup members as the culprit based on guessing with probability *g*. The culprit is then selected among the fillers with probability 1 ÷ lineup size (e.g., 1 ÷ 6 in a six-person lineup). With probability 1 – (1 ÷ lineup size), one of the fillers is selected (e.g., 5 ÷ 6 in a six-person lineup). The probability with which guessing leads to a culprit identification thus depends only on the lineup size and not on any cognitive process which is why this element of the model is a known constant and not a parameter that has to be estimated from data. In the present context it is important to realize that this constant is the same irrespective of whether first-yes-counts instructions are used, provided a proper randomization procedure is in place. Specifically, if an eyewitness selects one person out of *N* persons in the lineup based on guessing, then the sampling probability that the selected person, out of the *N* persons in the lineup, is the culprit is always 1 ÷ *N*, irrespective of the presence or absence of first-yes-counts instructions. If none of the lineup members is selected based on guessing, which occurs with probability 1 – *g*, then the lineup is falsely rejected.

**Figure 1:**
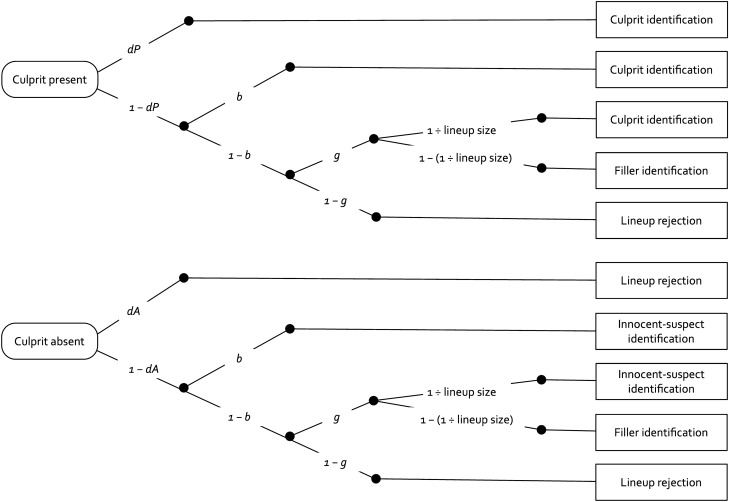
Graphical illustration of the 2-HT eyewitness identification model. The rounded rectangles on the left represent lineups in which the culprit is either present or absent. The rectangles on the right represent the possible responses of an eyewitness in a culprit-present or a culprit-absent lineup. The letters attached to the branches represent the probabilities of the latent cognitive processes postulated by the model that lead to the responses of the eyewitness (*dP*: detection of the presence of the culprit; *b*: biased selection of a suspect who stands out from the fillers; *g*: guessing-based selection among the lineup members; *dA:* detection of the absence of the culprit). The *lineup size* is a known constant: the number of persons in the lineup.

The lower tree of the model represents the processes that occur in culprit-absent lineups. The absence of the culprit and the fact that no one else in the lineup can possibly be the culprit is detected with probability *dA*, leading to the correct rejection of the lineup. With probability 1 – *dA*, the absence of the culprit is not detected, in which case the same non-detection-based processes occur with the same probabilities as in culprit-present lineups, the difference being that guessing and biased selection in culprit-absent lineups may lead to the selection of the innocent suspect rather than to the selection of the culprit (see^34^ and^35^ for more details).

Here we focus on the culprit-presence-detection parameter *dP* which is conceptually similar, but not identical, to the discrimination between culprits and innocent suspects that is hypothesized to be reflected in the partial area under the curve in analyses based on receiver operating characteristics and on the guessing-based-selection parameter *g* which provides a single, validated and unambiguous measure of the selection of lineup members based on guessing.

In a first step, the 2-HT eyewitness identification model was used to reanalyze the sequential-lineup data reported by Horry et al.^32^ to assess the effects of the first-yes-counts instructions on culprit-presence detection and guessing-based selection within a single coherent and experimentally validated measurement model. In addition to this reanalysis, an independent conceptual replication of the sequential-lineup conditions of Horry et al.^32^ was conducted, the data of which were also analyzed using the 2-HT eyewitness identification model. The central conclusion drawn from the analyses of Horry et al.^32^ was that the first-yes-counts instructions left detection-based processes unaffected but decreased the participants’ willingness to make identification decisions. If this conclusion is valid, then the first-yes-counts instructions should not affect the probability with which the presence of the culprit is detected whereas the probability of guessing-based selection should be lower in the condition with first-yes-counts instructions than in the condition without such instructions. In other words, the culprit-presence-detection parameter *dP* of the 2-HT eyewitness identification model should not differ between the condition with first-yes-counts instructions and the condition without such instructions whereas the guessing-based-selection parameter *g* of the 2-HT eyewitness identification model should be significantly lower in the condition with first-yes-counts instructions than in the condition without such instructions.

## Reanalysis of the sequential-lineup data reported by Horry et al.^32^

Horry et al.^32^ randomly assigned 896 participants to the conditions of a 3 (lineup type: sequential lineup with first-yes-counts instructions vs. sequential lineup without such instructions vs. simultaneous lineup) × 2 (culprit presence: culprit present vs. culprit absent) × 2 (suspect position in the lineup: 2 vs. 5) between-subjects design. Here we focus on the effect of the first-yes-counts instructions. Therefore, we reanalyzed the identification decisions of the 559 participants in the two sequential-lineup conditions (with first-yes-counts instructions and without such instructions), aggregated across suspect position.

### Method

Participants watched one out of four possible mock-crime videos with the same content but a different culprit. Lineups were composed of one suspect and five fillers who matched the description of the suspect. In culprit-present lineups, the suspect was the culprit from the one video that had been seen. In culprit-absent lineups, the suspect was a culprit from one of the other videos that had not been seen. Participants were asked to identify a culprit in one lineup but they knew that the culprit did not have to be in the lineup. The instructions were identical for all participants except that the participants in the condition with first-yes-counts instructions received the following additional information: “If you respond ‘yes’ to a photo, you will not be able to change that decision, and you will not be able to respond ‘yes’ to any later photos”^32^. Independent of the instructions, participants were able to make multiple identification decisions in both sequential-lineup conditions and if they did, they were presented with a second lap of the lineup to clarify their decision. In the condition with first-yes-counts instructions, each participant’s first identification decision in the initial lineup was used for the analyses. In the condition without such instructions, this was different. If participants made multiple identification decisions in the initial lineup, the unique identification decision given in the second lap was used for the analyses. For more details, see Horry et al.^32^.

### Results

For all analyses reported in this article, multiTree^39^ was used to obtain parameter estimates and to perform likelihood-ratio goodness-of-fit tests for which the α level was set to 0.05. The response frequencies underlying the analyses reported here are shown in Table 1. These response frequencies were taken from the penultimate and the last lines of Horry et al.’s^32^ Table 3 in which the data are already aggregated across suspect position.

**Table 1:** Response frequencies from Table 3 of Horry et al.^32^.

	Culprit-present lineups			Culprit-absent lineups		
	Culprit identifications	Filler identifications	Lineup rejections	Innocent-suspect identifications	Filler identifications	Lineup rejections
First-yes-counts instructions	71	26	61	7	38	102
No first-yes-counts instructions	69	23	30	5	48	79

**Figure 2:**
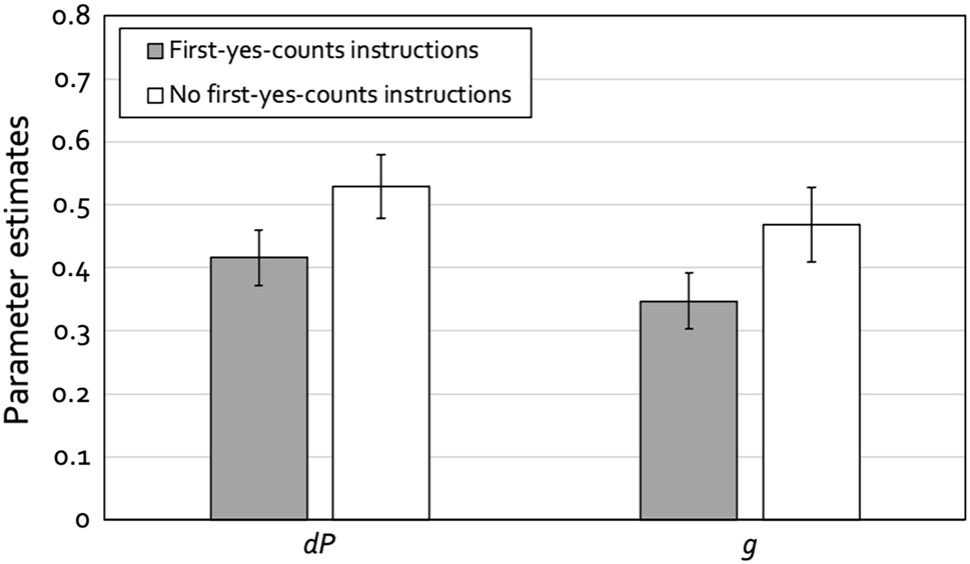
Estimates of parameters *dP* (detection of the presence of the culprit) and *g* (guessing-based selection) of the 2-HT eyewitness identification model as a function of lineup instructions (first-yes-counts instructions vs. no first-yes-counts instructions) for the sequential-lineup data of Horry et al.^32^. The error bars represent the standard errors.

We needed two instances of the model depicted in Fig. 1, one for the condition with first-yes-counts instructions and one for the condition without such instructions. Given that the lineups comprised six persons, the sampling probability with which guessing-based selections lead to identifications of the suspect, given by 1 ÷ lineup size, was set to 0.16667 as an approximation to 1 ÷ 6. We wanted to begin with a base model that was as simple as possible. Therefore, we used whatever we could derive from the design of the study to impose restrictions onto the 2-HT eyewitness identification model. First, given that the same, well-constructed lineups were used in both conditions (which were essentially fair, see below), we set the biased-suspect-selection parameter *b* to be equal across the two instruction conditions. Second, for the same reason the culprit-absence-detection parameter *dA* was also set to be equal across the two conditions. The base model incorporating these two restrictions fit the data, *G*^2^(2) = 2.42, *p* = 0.298. The estimates of parameters *b* and *dA* were 0.00 (*SE* = 0.02) and 0.13 (*SE* = 0.12), respectively. The estimates of the culprit-presence-detection parameter *dP* and of the guessing-based-selection parameter *g* are displayed in Fig. 2.

In multinomial processing-tree models, hypotheses can be tested directly at the level of the postulated processes. For instance, the hypothesis that the culprit-presence-detection parameter *dP* does not differ between the condition with first-yes-counts instructions and the condition without such instructions can be implemented by setting parameter *dP* to be equal between these conditions. If the model including this equality restriction provides a significantly worse fit to the data than the base model, then it is necessary to conclude that parameter *dP* differs between conditions. Parameter *dP* did not differ significantly between the condition with first-yes-counts instructions and the condition without such instructions, *ΔG*^2^(1) = 3.05, *p* = 0.081. In contrast, parameter *g* was significantly lower in the condition with first-yes-counts instructions than in the condition without first-yes-counts instructions, *ΔG*^2^(1) = 5.31, *p* = 0.021.

### Discussion

The results of this reanalysis are compatible with the conclusions reached by Horry et al.^32^. The presence or absence of first-yes-counts instructions did not affect the probability with which the culprit was detected whereas it affected the probability of guessing-based selection in the expected direction. Specifically, the culprit-presence-detection parameter *dP* of the 2-HT eyewitness identification model did not differ significantly between the condition with first-yes-counts instructions and the condition without such instructions whereas the guessing-based-selection parameter *g* of the 2-HT eyewitness identification model was significantly lower in the condition with first-yes-counts instructions than in the condition without such instructions.

However, at a descriptive level the culprit-presence-detection parameter *dP* was lower in the condition with first-yes-counts instructions than in the condition without such instructions. This observation may fuel the suspicion that there might in fact be a difference in *dP* which could turn out to be statistically significant if the relevant statistical test were even more sensitive. This was one reason as to why we aimed at replicating the sequential-lineup conditions of Horry et al.^32^ and to test, based on even more datapoints and thus with even more sensitivity, whether first-yes-counts instructions have indeed no effect on culprit-presence detection while decreasing the probability of guessing-based selections.

## Conceptual replication of the sequential-lineup conditions of Horry et al.^32^

The reliability of findings is important^33^ because conclusions can only be valid if they are based on reliable data. What is more, an independent replication of critical findings is regarded as the gold standard in assessing the reliability of findings and as a necessary step in science to increase the trust in new findings^40^. We therefore tested the reliability of the findings of Horry et al.^32^ in an independent conceptual replication of their sequential-lineup conditions. We chose to perform a conceptual replication because, if successful, a test of the replicability of findings is even more powerful if one can show that the core finding does not depend on details of the experimental implementation. Our implementation differed in the following aspects from that used in the study of Horry et al.^32^. First, in the condition with first-yes-counts instructions, only one identification was technically possible. Second, participants saw each lineup only once. Third, if multiple identifications occurred in the condition without first-yes-counts instructions, we considered the last decision as a revision of any previous decisions; therefore, the last identification was used in the analysis. Fourth, we used a multiple-culprit mock crime; every participant was presented with four different lineups, two of which were culprit-present lineups and two were culprit-absent lineups. Finally, the positions of all lineup members, including the culprit and the innocent suspect, were randomly selected. Given these differences between the two studies, a replication of the results of Horry et al.^32^ would indicate that their results are reliable and robust in the sense that they do not depend on the details of the experimental implementation. In this case, the culprit-presence-detection parameter *dP* of the 2-HT eyewitness identification model should not differ between the condition with first-yes-counts instructions and the condition without such instructions whereas the guessing-based-selection parameter *g* should be significantly lower in the condition with first-yes-counts instructions than in the condition without such instructions.

### Method

#### Sample

Participants were recruited using the Cint research panel (https://www.cint.com). Criteria for participation were legal age (≥ 18 years), good eyesight and German language skills. Given that the design of the present experiment comprised two groups, we aimed at recruiting about half as many participants as in the four-group experiments of Winter et al.^35^ in which about 750 participants were recruited. We thus aimed at collecting about 375 data sets and terminated the data collection at the end of the day at which this criterion was surpassed. Of the 421 participants who gave informed consent, 52 did not complete the study. Another 14 participants were excluded because they had failed to respond correctly to the attention check question, because they had indicated technical problems or because they had participated more than once which implies that they had watched the mock-crime video more than once. The final sample comprised 355 participants (142 women, 210 men, 3 diverse) with a mean age of 46 years (*SD* = 15). A sensitivity analysis using G*Power^41^ showed that given α = β = 0.05, a sample size of *N* = 355 and four identification decisions per participant, it was possible to detect an effect of the first-yes-counts instructions of size *w* = 0.10 on the model parameters.

#### Ethics statement

All participants gave informed consent prior to beginning the experiment. They were informed that during the experiment they would see a video in which a group of persons would attack another person verbally and physically. Participants were asked not to participate if they felt uncomfortable when anticipating to watch such a video. Ethical approval had been obtained from the ethics committee of the Faculty of Mathematics and Natural Sciences of Heinrich Heine University Düsseldorf for a series of experiments to which the current experiment belongs. The experiment was run in accordance with the declaration of Helsinki.

#### Materials and procedure

Materials and procedure were essentially the same as those of Winter et al.^35^ with the exception of the manipulation of the presence or absence of the first-yes-counts instructions that are described in detail below. The experiment was run online using SoSci Survey^42^. It was possible to participate with a computer and a laptop but not with a mobile device such as a smartphone. Participants were asked to complete the study alone and in a quiet environment and to put their browser into full-screen mode prior to starting the experiment.

##### Mock-crime videos

Participants watched one of two mock-crime videos. In each of the two videos, four alleged hooligans of the German soccer club FC Bayern München—henceforth referred to as the culprits—attacked an alleged fan of a rivaling soccer club Borussia Dortmund—henceforth referred to as the victim—at a bus station. The culprits and the victim wore typical fan clothing (shirts, scarfs and caps) of their respective soccer clubs. The culprits insulted the victim, poked fun at him and tossed his belongings around. At the end of the video, the victim got knocked to the ground. The culprits continued to physically abuse the victim before they apparently noticed another person approaching (not visible in the video) and ran away shouting loudly. The two videos (henceforth Video 1 and Video 2) showed the same events (i.e., the same verbal abuse and the same acts of violence in the same sequence and with the same timing), but the victims and the culprits were played by different actors. However, the victim in Video 1 matched the victim in Video 2 in terms of age, hair color, hair style and stature, as determined by the present authors. The same was true for the four culprits, that is, Hooligan A in Video 1 matched Hooligan A in Video 2, Hooligan B in Video 1 matched Hooligan B in Video 2 and so on. It was randomly determined which of the two parallel versions of the video was shown to a particular participant. The videos were about 130 s long and shown in a resolution of 885 × 500 pixels.

Participants started the video by clicking on a “Start” button. Participants could not stop, fast forward or replay the video. Once the video had ended, participants were asked to answer an attention check question probing for the type of persons seen in the video. The correct response was to select “soccer fans” from a list of ten alternatives.

##### Lineup procedures

Participants were informed that they had to identify the FC Bayern München hooligans from the video they had just seen. Participants received two-sided lineup instructions that emphasized both the need to identify the culprit if the culprit was present and the need to reject the lineup if the culprit was absent. Participants were not informed about how many lineups were about to follow. Each lineup consisted of the facial photographs of six persons, one culprit or innocent suspect and five fillers. Participants saw two culprit-present lineups and two culprit-absent lineups.

To manipulate the suspect’s guilt, the crossed-lineup procedure^35^ was used. In each of the two culprit-present lineups, a randomly selected face of one of the hooligans of the video the participants had seen was presented among the fillers. In each of the two culprit-absent lineups, the face of an innocent suspect was presented that the participants had not witnessed committing a crime. The innocent suspect was one of the hooligans from the video that the participants had not seen. For instance, if participants had seen Video 1, two randomly selected hooligans (e.g., Hooligan B and Hooligan C) from Video 1 served as the culprits in the two culprit-present lineups, while two of the hooligans from Video 2 (Hooligan A and Hooligan D in this example) served as the innocent suspects in the culprit-absent lineups. The same set of fillers was used in culprit-present and culprit-absent lineups. For each of the four lineups, five photographs of male adults aged between 18 and 29 years were selected from the Center for Vital Longevity Face Database of Minear and Park^43^ to serve as fillers. The fillers resembled the culprit and the matching innocent suspect in age, hair color, hair style and stature. Together with the fact that it was randomly determined whether participants saw Video 1 or Video 2, the crossed-lineup procedure ensures that the culprits and innocent suspects differ to the same degree, on average, from the fillers in the lineup. The crossed-lineup procedure is ecologically valid because the situation it creates corresponds closely to that of a real-world lineup in which the photograph of the suspect—whose status as culprit or innocent suspect is unknown to the police—stems from a different source (e.g., social media) than the photographs of the fillers (e.g., a database). Therefore, the photographs of the fillers may differ to some degree from the photograph of the suspect in terms of variables such as tint, resolution or softness, particularly in cases in which the photographs of the fillers are digitally manipulated^31,44–46^. All photographs showed the faces from a frontal view with a neutral facial expression against a black background with no clothes visible. The photographs were edited to harmonize face sizes and lighting conditions and were presented in a resolution of 142 × 214 pixels. The order of the lineups was randomized, as was the position of the culprit or innocent suspect and the fillers in each lineup.

Participants were randomly assigned either to the condition with first-yes-counts instructions or to the condition without such instructions. In the condition with first-yes-counts instructions, each lineup was preceded by a screen announcing the next lineup as the “1st lineup”, “2nd lineup” and so on and the following statement (the following quotations are translations of text originally presented in German): “If you choose ‘Yes, was present’ for one face, this decision is irrevocable. You will not be able to select ‘Yes was present’ for any other face in the lineup”. A click on the “I am ready” button started the successive presentation of the photographs. Participants decided for each photograph whether it showed one of the culprits or not by clicking on the “Yes, was present” button or the “No, this person was not present” button, respectively. Either response initiated the presentation of the next photograph. Immediately after participants’ first and only identification decision in a particular lineup, the ‘Yes, was present’ button was disabled and its label changed to ‘A yes response is no longer possible’ for all remaining photographs of the lineup for which participants were only able to select the ‘No, this person was not present’ button.

In the condition with no first-yes-counts instructions, each lineup was preceded by a screen announcing the next lineup as the “1st lineup”, “2nd lineup” and so on, without the first-yes-counts statement. A click on the “I am ready” button started the successive presentation of the photographs. Within each lineup, participants decided for each photograph whether it showed one of the culprits or not by clicking on the “Yes, was present” button or the “No, this person was not present” button, respectively. Either response initiated the presentation of the next photograph. In case of more than one identification decision in a lineup, the last decision was used in the analysis.

A lineup was counted as rejected if none of the members of the lineup was identified as one of the culprits. To make the procedure similar to the procedure used by Horry et al.^32^ and to that of a typical police lineup, participants also indicated how confident they were that their decision was correct. After every lineup, participants had to click on a “Continue” button to start the next lineup or, after the final lineup, to reach a screen on which questions were asked about possible technical difficulties during the online experiment. At the end of the experiment, all participants were informed that the crime shown in the video had been staged and they were informed about the purpose of the experiment.

### Results

**Figure 3:**
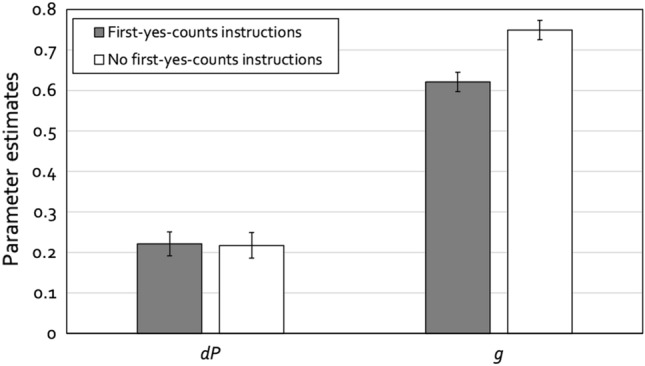
Estimates of parameters *dP* (detection of the presence of the culprit) and *g* (guessing-based selection) of the 2-HT eyewitness identification model as a function of lineup instructions (first-yes-counts instructions vs. no first-yes-counts instructions) for the conceptual replication of the sequential-lineup conditions of Horry et al.^32^. The error bars represent the standard errors.

The response frequencies underlying the analysis reported here are shown in Table 2. We needed two instances of the model depicted in Fig. 1, one for the condition with first-yes-counts instructions and one for the condition without such instructions. Given that the lineups comprised six persons, 1 ÷ lineup size was set to 0.16667 as an approximation to 1 ÷ 6. As before, we wanted to begin with a base model that was as simple as possible. The design of the present experiment makes it possible to derive the same restrictions for the 2-HT eyewitness identification model that we derived from the design of the experiment of Horry et al.^32^ in the reanalysis presented above. The biased-suspect-selection parameter *b* and the culprit-absence-detection parameter *dA* were each set to be equal across the two conditions. The base model incorporating these restrictions fit the data, *G*^2^(2) = 3.09, *p* = 0.214. The estimates of parameters *b* and *dA* were 0.01 (*SE* = 0.01) and 0.06 (*SE* = 0.04), respectively. The estimates of the culprit-presence-detection parameter *dP* and the guessing-based-selection parameter *g* are displayed in Fig. 3.

**Table 2:** Response frequencies obtained in the conceptual replication of the sequential-lineup conditions of Horry et al.^32^.

	Culprit-present lineups			Culprit-absent lineups		
	Culprit identifications	Filler identifications	Lineup rejections	Innocent-suspect identifications	Filler identifications	Lineup rejections
First-yes-counts instructions	112	147	107	31	184	151
No first-yes-counts instructions	110	167	67	50	194	100

Parameter *dP* did not differ significantly between the condition with first-yes-counts instructions and the condition without such instructions, *ΔG*^2^(1) = 0.01, *p* = 0.936. In contrast, parameter* g* was significantly lower in the condition with first-yes-counts instructions than in the condition without such instructions, *ΔG*^2^(1) = 21.16, *p* < 0.001.

### Discussion

The results of this conceptual replication of the sequential-lineup conditions of Horry et al.^32^ nicely fit with the results of the original study. The presence or absence of first-yes-counts instructions did not affect the probability with which the culprit was detected whereas guessing-based selection was significantly less likely to occur in the condition with first-yes-counts instructions than in the condition without such instructions. This confirms the conclusions drawn from the reanalysis of the data reported by Horry et al.^32^. The fact that there was again no difference between conditions in parameter *dP* may be seen as particularly remarkable given that the relevant statistical test here was based on more data points (cf. Tables 1 and 2) and thus more sensitive than the corresponding test in the reanalysis of the data reported by Horry et al.^32^.

## General discussion

Horry et al.^32^ were the first to demonstrate experimentally that the presence or absence of first-yes-counts instructions in sequential lineups affects eyewitness identification decisions. In order to assess the reliability of the sequential-lineup findings of Horry et al.^32^ and to deal with the three threats to the validity of their conclusions explicated in the introduction above, we reanalyzed their data using the 2-HT eyewitness identification model and conducted a conceptual replication of the sequential-lineup conditions of their experiment. The results of both the reanalysis and the conceptual replication are well in line with the conclusions that were drawn from the analyses of Horry et al.^32^: First-yes-counts instructions in sequential lineups do not affect the ability of eyewitnesses to detect the culprit but first-yes-counts instructions decrease the probability of guessing-based selection compared to instructions in which it is not mentioned that only the first identification decision counts. This conclusion was reached here using a single coherent and experimentally validated measurement model in which the complete 2 × 3 data structure of eyewitness identification decisions is used to separately measure the detection and guessing processes involved in eyewitness identification decisions. On the one side, we regard using a single coherent measurement model as advantageous compared to the use of two separate and conceptually different measurement models by Horry et al.^32^—receiver operating characteristics for eyewitnesses’ ability to discriminate between culprits and innocent suspects and an implicit ad-hoc measurement model for eyewitnesses’ response criterion based on both the suspect identification rate and the lineup rejection rate. On the other side, it seems quite impressive that two such different approaches to measuring the processes underlying eyewitness identification decisions lead to the same basic conclusions. Not only did the two measurement approaches differ, but the identification conditions in Horry et al.^32^ and the replication study also differed in many respects such as in the number of culprits, the length of the mock-crime video and the setting in which participants were tested (online vs. laboratory). As an aside, these procedural differences may be the reason why the probability of culprit-presence detection was lower and the probability of guessing-based selection was higher in the present conceptual replication than in the reanalysis of the Horry et al.^32^ data. However, the important point is that the relevant comparisons between the conditions with and without first-yes-counts instructions led to the same conclusions irrespective of these procedural changes. The conclusion about the effect of first-yes-counts instructions on the processes underlying eyewitness identification decisions thus do not depend on the details of the experimental implementation.

With respect to guessing-based selection, the conclusion drawn from both datasets analyzed here is that first-yes-counts instructions serve to reduce the probability with which this process occurs. Should one thus use first-yes-counts instructions if the goal is to reduce guessing-based selection? There is in fact a far simpler and more straightforward method for reducing the probability of guessing-based selection without affecting the probability of culprit-presence detection. As Winter et al.^35^ have shown in their Experiment 3, using pre-lineup instructions that discourage all but the very certain identifications suffices to reach this goal.

Given that the lineups used in both the experiment of Horry et al.^32^ and the present conceptual replication were essentially fair (parameter *b* was essentially zero in both conditions in both analyses), there was no basis for biased-suspect selection to differ between the condition with first-yes-counts instructions and the condition without such instructions. Therefore, the existing data do not allow us to draw firm conclusions about whether, and if so how, first-yes-counts instructions might affect biased-suspect selection in unfair lineups. The effect of first-yes-counts instructions on biased-suspect selection in unfair lineups is thus an issue for future research to explore.

Knowing that and how first-yes-counts instructions affect the processes underlying eyewitness identification decisions is important. This is so because one may now conclude that a sizeable number of previous eyewitness identification studies indeed lack ecological validity in that they underestimate the tendency of eyewitnesses to select the culprit based on guessing relative to real police lineups (e.g.,^10–27,47^). Parallel to what Steblay et al.^8^ have stated, we know of no jurisdiction that has implemented a rule to take into account only the witnesses’ first identification decision in sequential lineups^3–5,29–31^. The reason as to why such a rule is avoided seems obvious. Legal systems typically come with a regulation as to how many fillers have to be in a lineup together with the suspect. For instance, in the United States a lineup usually consists of five fillers and the suspect^48^, in the United Kingdom a lineup usually consists of eight fillers and the suspect^29^ and in Germany a lineup usually consists of seven fillers and the suspect^2^. A first-yes-counts rule could be seen to undermine these regulations. Specifically, whenever an eyewitness would make an identification decision prior to seeing the last photo in the lineup and thus terminate the lineup presentation, it could be argued by defense lawyers that the actual lineup size was lower than the lineup size stipulated by the legal system. The most straightforward solution to the problem is to assume that if multiple identification decisions occur, later identification decisions imply a revision of earlier identification decisions which implies that the last identification decision in a lineup is the one to be used. Alternatively, in some legal systems eyewitnesses are obliged to perform multiple laps through a lineup before they are allowed to make one final identification (e.g.,^3,29^). Either way, research on sequential lineups needs to implement one of the rules instead of the first-yes-counts rule if the aim is not to decrease the ecological validity of this research.

## Data Availability

The datasets generated and analyzed during the current study are available in the OSF repository, https://osf.io/pxhk8/.
